# Father-Child and Mother-Child Interactions with Children with Anxiety Disorders: Emotional Expressivity and Flexibility of Dyads

**DOI:** 10.1007/s10802-017-0271-z

**Published:** 2017-02-07

**Authors:** Daniëlle Van der Giessen, Susan Maria Bögels

**Affiliations:** Department of Child Development and Education, Research Priority Area Yield, University of Amsterdam, P.O. Box 15780, 1001 NG Amsterdam, The Netherlands

**Keywords:** Emotion dynamics, Parent-child interactions, Child and parental anxiety, State space grids

## Abstract

This observational study examined whether emotional expressivity and emotional flexibility differed between parent-child dyads with and without children with an anxiety disorder (AD). Effects of parents’ own AD on emotional expressivity and flexibility of dyads was also studied. The sample consisted of 128 referred children (59.4% girls) with an AD (8–18-year-olds) and both of their parents, and 44 matched non-AD children (63.6% girls) and both of their parents. Father-child and mother-child dyads were videotaped while discussing a conflict. Measures of dyadic emotional expressivity (positive and negative affect) and dyadic emotional flexibility (transitions, dispersion, average duration) were derived from these interactions using state space grid analysis. No differences existed in emotional expressivity of parent-child dyads with or without AD children, however both father-child and mother-child dyads with a child with an AD displayed less emotional flexibility during interactions than healthy controls. Mother-child dyads where both mother and child had AD showed more emotional expressivity and less emotional flexibility compared to mother-child dyads with only AD children and to dyads without AD. In particular, the inability to flexibly move in and out of different emotions distinguishes healthy dyads from non-healthy dyads. Targeting emotional flexibility of dyads with children with an AD, and also emotional expressivity of dyads when mother has an AD, might be a valuable goal for family-based intervention.

Giving the impairing nature of anxiety, considerable research in the past decade has been devoted to understanding anxiety disorders (AD) of children. Accumulating evidence shows that environmental factors are more strongly related to children’s anxiety than genetic factors (Eley et al. 2015; Morris et al. 2007). Most research on child anxiety has focused on parenting during interactions, such as parental overprotection (Bögels and Brechmann-Toussaint 2006), but the associations between child anxiety and such parenting factors are modest (see the meta-analyses of McLeod et al. 2007; Van der Bruggen et al. 2008). Less research has been conducted on other aspects of parent-child interactions, such as emotion regulation, but a few findings are beginning to emerge. Children with an AD are found to have difficulty expressing and managing anger and sadness and perceive themselves as less able to successfully manage emotionally provocative situations (e.g., Suveg and Zeman 2004). Also, parents of children with an AD tend to encourage the suppression of emotional expression, and express less positive and more negative emotions themselves (Hudson et al. 2008; Suveg et al. 2008). While, emotion regulation during interactions is considered to be a dynamic process to which both parent and child contribute (Butler 2011; Fogel 1993; Morelen and Suveg 2012), most research examined children’s and parents’ emotion regulation skills at an individual level and used static measures (e.g., questionnaires or global rating coding systems). Although informative, this measurement approach fails to capture the dynamic, interconnected and contextually specific emotions that children and parents together employ during real-life interactions (Butler 2011; Frijda 2007). Thus, despite growing evidence on the interconnected nature of children’s and parents’ emotions during interactions as well as research showing the link between parent-child emotion regulation difficulties and children’s AD, relatively little research examined this in tandem. Investigating at a dyadic level how parent-child dyads express and adjust emotions in real-time during interactions might have crucial relevance for more effective treatment of children’s AD.

Emotional expressivity of parent-child dyads is thought to be an important indicator of adaptive socio-emotional functioning (Morris et al. 2007). Emotionally expressive parent-child interactions, characterized by more positive affect and moderate levels of negative affect, might indicate that emotion expression is acceptable and valued in these dyads. In contrast, inexpressive or highly negative emotional interactions might reflect a discouraging emotional climate, potentially reflecting inadequate emotional functioning of dyads. Families with AD children are thought to have difficulties appropriately expressing positive and negative affect in emotionally evocative situations (Hannesdottir and Ollendick 2007; Suveg et al. 2010). This might partly be because a central component of children’s AD is the predominance and high intensity of negative emotional experiences as well as hyperarousal, which might lead to either over-control of emotions (i.e., suppression) or under-control of emotions (i.e., more negative affect) in emotionally arousing interactions (Suveg and Zeman 2004). On the other hand, parents of children with an AD might also be afraid to express negative emotions, as they may underestimate the emotion regulation abilities of their child with an AD and perceive the child as extremely vulnerable, thereby discouraging the expression of emotions (Bögels and Brechman-Toussaint 2006).

Existing observational studies indeed showed that difficulties with emotion expression during parent-child interactions are related to children’s AD. During emotion discussions, mothers of children with an AD expressed less positive emotions than mothers of non-AD children and discouraged the discussion of negative emotional experiences (Suveg et al. 2005). When including both fathers and mothers in emotion discussions with the child, similar emotional patterns were found showing that parents of children with an AD exhibited less positive affect during the discussions than parents of children without an AD (Hudson et al. 2008). Suveg et al. (2008) showed that mothers and fathers of boys with an AD, not girls, exhibited less positive affect and more negative affect during emotion discussions than did fathers and mothers of boys without an AD. Finally, children with an AD displayed less positive emotions during the emotional discussions than non-AD children (Hudson et al. 2008; Suveg et al. 2008).

Together, research shows deficits on an individual level in emotional expressivity during parent-child interactions when children have an AD. Particularly, when children have an AD, children and parents showed diminished positivity when dealing with emotionally negative experiences. What we do not understand yet is how individual emotional expressivity (i.e., of parents and children) combines in a dyadic context, and how it is related to child anxiety. Emotions during interactions are embedded in a relational context (Butler 2011), and parents’ and children’s emotional expressions appear to be reciprocally related (Morelen and Suveg 2012). Vital information about the dyadic and interrelated nature of emotions might be missing when examining parents’ and children’s emotional expressivity during interactions in isolation from each other. Therefore, a critical question remains whether there are differences between parent-child dyads with AD children and non-AD children in levels of dyadic emotional expressivity (i.e., dyadic positive and negative affect).

Emotional flexibility of parent-child dyads is considered to be a hallmark of healthy functioning (Butler 2011; Granic 2005). All emotions, including negative ones, are thought to be adaptive, and important to express in appropriate contexts (Gross 2007). The extent to which dyads together can control and adjust their emotions according to situational demands is important for emotional functioning. This means that some degree of negativity of parent-child dyads during conflict interactions is appropriate, as long as dyads can also switch to positive emotions, thereby managing their emotions effectively. It also suggests that parent-child dyads that only express positive emotions and suppress negative emotions during conflict interactions, seem unable to flexibly adapt their emotional responses according to the emotional demands of such contexts. As such, being stuck in either positive or negative emotions, is what is thought to be problematic, even more so than the average amount of positive or negative emotions expressed during interactions (Granic 2005; Houben et al. 2015). This idea has led researchers to hypothesize that parent-child dyads with high emotional flexibility, who are able to flexibly shift in and out of a wide range of dyadic emotions as the situation warrants, are adequately regulating their emotions, which is related to health and well-being (Granic 2005). In contrast, parent-child dyads with low emotional flexibility, that have a tendency to get stuck in specific dyadic emotions during interactions, thereby staying in a limited emotional repertoire for longer periods of time, are thought to have poor control over their emotions, and it is expected to be associated with psychopathology, including anxiety. Due to the high number of intense negative experiences and hyperarousal associated with anxiety, children with an AD (and their parents) often are characterized by a relatively stable, small and stereotyped emotional repertoire, extending beyond anxiety-provoking situations (Cisler et al. 2010; Kashdan and Rottenberg 2010). When confronted with challenging situations, parent-child dyads with children with an AD might not have the resources and skills available to search together for alternative ways of responding, which may inhibit emotional flexibility of parent-child dyads during interactions.

Emotional inflexibility (i.e., rigidity) of parent-child dyads has been associated with children’s emotional functioning. More emotional rigidity of parent-child dyads has been related to more internalizing and externalizing problems in high risk children in kindergarten (Hollenstein et al. 2004). During a challenging puzzle task at age 3, less emotional flexibility of father–child dyads in particular predicted more externalizing problems at age 5 (Lunkenheimer et al. 2011). In a community sample of mother-adolescent dyads, less emotional flexibility of mother-child dyads during conflict interactions in early adolescence predicted more anxiety and depressive symptoms of adolescents 5 years later (Van der Giessen et al. 2015). Finally, increases over the course of treatment in emotional rigidity of parent-child dyads was associated with less improvement of children’s aggressive behavior after treatment (Granic et al. 2007). Although theory and research provides support for an association between emotional rigidity of parent-child dyads and externalizing and internalizing problems, it is unknown whether a lack of emotional flexibility in parent-child dyads is also associated with clinical levels of child anxiety. To advance research on child AD and to inform intervention efforts it is important to examine emotional flexibility as a dyadic process that unfolds in the moment, and to investigate differences in flexibility between parent-child dyads with and without AD children.

Not only children’s, but also parents’ own ADs have been associated with a more negative emotional family climate (Bögels and Phares 2008). Parents with an AD have been found to display more dysfunctional emotional reactions during interactions (Teetsel et al. 2014; Van der Bruggen et al. 2008), and this appears to hold for AD fathers with AD children in particular (Bögels et al. 2008; Hudson et al. 2008). Like children with an AD, parents with an AD might also not possess adaptive resources for expressing and managing positive and negative emotions, thereby further contributing to less adaptive emotional patterns of parent-child dyads (Morris et al. 2007). It might therefore be particularly detrimental to the emotional dynamics of interactions when both the parent as well as the child have an AD. Nevertheless, knowledge is lacking on how parental AD, in addition to children’s AD, is associated with dyadic emotional expressivity and dyadic emotional flexibility during parent-child interactions. Finally, while fathers’ contribution and role during interactions with children might be different from that of mothers, particularly when fathers have an AD themselves (Bögels and Phares 2008; Lunkenheimer et al. 2011; Morris et al. 2007; Suveg et al. 2008), studies mostly examined emotional expressivity and flexibility of mother-child dyads. Therefore, there is a great need for observational research exploring differences in emotional flexibility between mother-child and father-child dyads with and without AD children.

To conclude, although knowledge on emotion regulation difficulties within families with an AD is growing, it is of great importance to gain more insight into dyadic emotional processes of parent-child dyads unfolding in the moment that are related to child and parent AD. This observational study tries to understand differences in dyadic emotional expressivity and dyadic emotional flexibility between parent-child dyads with and without AD children. Differences between father-child dyads and mother-child dyads were investigated as well as the effects of parents’ AD on dyadic emotional expressivity and dyadic emotional flexibility. Regarding expressivity, we expected that parent-child dyads with AD children would particularly show less positive emotions, but also more negative emotions during interactions than parent-child dyads with non-AD children. Regarding flexibility, we expected that parent-child dyads with AD children would display less emotional flexibility during interactions than dyads with non-AD children. Considering that only little research to date has systematically addressed differences between father-child and mother-child dyads in these dyadic emotional processes, we explored whether emotional expressivity and flexibility differed between mother-child and father-child dyads with and without AD children. To further narrow and enhance our understanding of group differences in dyadic emotional processes, we investigated the effects of parents’ AD on emotional expressivity and flexibility in parent-child interactions.

## Method

### Participants and Procedure

In the current study 128 children with an AD and 44 matched children without an AD as well as their families participated. The AD group consisted of children referred by their general practitioner to one of eight community mental health centers in the Netherlands because of a primary AD, and were participating in a randomized clinical trial comparing child-focused and family-focused Cognitive Behavior Therapy (Bodden et al. 2008). Inclusion criteria were age 8 to 18 years, a primary anxiety disorder (no obsessive compulsive disorder or post-traumatic stress disorder as primary disorder, in line with DSM-5), IQ ≥ 80, and at least one parent willing to participate. Children were excluded when they suffered from substance abuse, current suicide attempts, untreated attention deficit hyperactivity disorder, pervasive developmental disorders, or psychosis. They were also excluded when they used anxiety-reducing medication, unless they kept a constant dosage or ended the use before start of the study. The non-AD group consisted of children without an AD and were recruited through advertisements in journals and magazines. The non-AD children were matched to the AD children based on children’s age, gender, and school type, in order to make the non-AD children comparable to the AD children on personal characteristics. Families received a € 50 fee. Medical-ethical approval was obtained, and all families signed informed consent. Where a biological and a step-parent were available, the biological parent was invited to participate if that parent had regular contact with the child. See Table 1 for participants’ personal characteristics.

**Table 1:** Characteristics of families with AD children and non-AD children

	AD children (*N* = 128)	Non-AD children (*N* = 44)	Effect size^a^
Characteristics			
Number (%) of girls	76 (59.4)	28 (63.6)	0.04
Age child (*M, SD*)	12.44 (2.7)	12.41 (2.6)	0.00
Primary school (*n*, %)	67 (52.3)	19 (43.2)	0.08
High school (*n*, %)	61 (47.7)	25 (56.8)	
Married families (*n*, %)	105 (82)	31 (70.5)	0.15
Age			
Mother (*M, SD*)	41.8 (4.82)	43.25 (5.26)	0.23
Father (*M, SD*)	44.95 (5.12)	44.96 (5.04)	0.02
Educational level^b^			
Mother (*M, SD*)	5.08 (1.99)	6.43 (1.4)	0.64***
Father (*M, SD*)	5.65 (2.04)	6.6 (1.92)	0.39**
Professional level^c^			
Mother (*M ,SD*)	3.9 (2.13)	4.36 (1.84)	0.20
Father (*M, SD*)	4.56 (2.00)	5.0 (2.0)	0.19
Primary AD			
Child			
Social phobia	41 (32%)		
Separation AD	34 (27%)	1 (2%)	
Generalized AD	23 (18%)		
Simple phobia	21 (16%)		
Agoraphobia and/or Panic disorder	9 (7%)	5 (11%)	
Mother			
Social phobia	12 (9%)		
Generalized AD	9 (7%)	3 (9%)	
Simple phobia	14 (11%)	2 (5%)	
Agoraphobia and/or Panic disorder	3 (2%)		
Father			
Social phobia	7 (5%)		
Generalized AD	3 (2%)		
Simple phobia	6 (5%)		
Agoraphobia and/or panic disorder	2 (2%)		

*AD* Anxiety Disorder

^a^ phi coefficient as an effect size for categorical variables, Cohen’s d as an effect size for continuous variables

^b^On a scale from 1(no education) to 9(university degree)

^c^On a scale from 1(labor for which no education is required) to 7(university degree required)

**p < 0.01, ***p < 0.001

### Measures

#### Children’s and Parents’ Anxiety Disorders

Children’s primary AD according to the DSM-IV was assessed with the child and parent Dutch version of the Anxiety Disorders Interview Schedule for Children (ADIS-C/P, Siebelink and Treffers 2001; Silverman and Albano 1996). In line with DSM-5, obsessive compulsive disorders and post-traumatic stress disorders were not diagnosed as a primary AD. Parents and children were interviewed separately by a clinician and each diagnosis was rated on a severity ranging from zero to eight (a score of four or more indicating a clinical diagnosis). According to ADIS instructions, child- and parent-reports were combined to determine diagnosis. Parents’ current AD was assessed with the adult ADIS-A (DiNardo et al. 1994). The ADIS-C/P and the ADIS-A have good psychometric properties (Brown et al. 2001; DiNardo et al. 1994; Silverman et al. 2001). The Interrater reliabilities for all ADIS diagnoses (kappa), based on the presence/absence of a diagnosis, were high; ADIS-C 0.89, ADIS-P 0.83, and ADIS-A 0.94 (Bodden et al. 2008). See Table 1 for children’s and parents’ primary anxiety diagnosis.

#### Parent-Child Interactions

All children participated in two dyadic 5-min videotaped conflict interactions, separately and in random order with their father and mother (Siqueland et al. 1996). The Issue Checklist (Robin and Weiss 1980) was used to determine the topic for father-child and mother-child interactions. It assesses how often dyads discussed 44 issues, such as doing homework, during the last 2 weeks, and how calm or angry the discussion was. Issues with the highest frequency and intensity ratings (average of dyad members’ ratings) were selected.

The conflict interactions of father-child and mother-child dyads were independently coded using the Simple Affect Coding system (SACS; Jabson et al. 2003), which has been applied successfully to parent-child interactions (Connell et al. 2011). SACS identifies the affect/emotions expressed during interactions through a combination of voice tone, facial expressions, and physical cues. Five mutually exclusive affect codes were used; positive affect, validation, anger/disgust, distress, and neutral. Codes were recorded continuously in real-time for each dyad member independently using The Observer XT9.0 (Noldus Information Technology 2009). Coders were intensively trained over a 3-month period to achieve a minimum inter-observer criterion of 75% agreement (0.65 kappa). Randomly, 20% of the videotapes were independently coded by 2 coders. Coders were unaware which sessions were used for observer agreement and were blind to parents’ and children’s diagnostic status. Using an event-unit based comparison with a 3 s tolerance window (Bakeman et al. 2009), the average inter-observer agreement was 85.22% (0.81 kappa) for father-child interactions with non-AD children and 81.71% (0.76 kappa) for father-child interactions with AD children, and 78.23% (0.73 kappa) for mother-child interactions with non-AD children and 80.13% (0.75 kappa) for mother-child interactions with AD children.

**Fig. 1:**
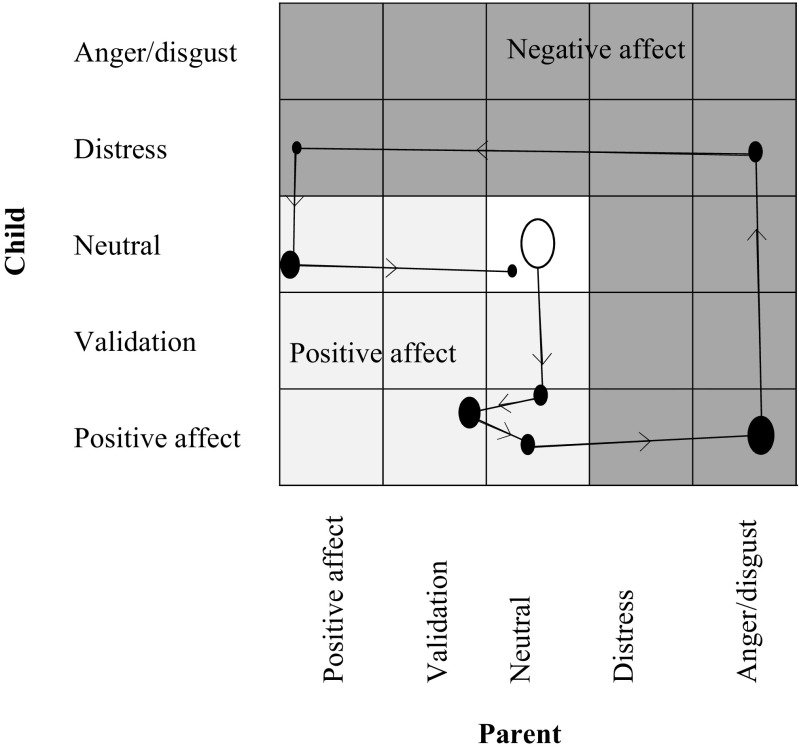
An example of a state space grid depicting a sequence of emotions of a parent-child dyad. The size of the circles is proportional to the duration of time each emotion is expressed, and the arrows reflect the changes between emotions. The light grey area on the grid is the positive affect region, the dark grey area on the grid is the negative affect region, and the white cell on the grid is the mutual neutral affect region. Separate grid were created for mother-child and father-child dyads

To capture emotional expressivity and flexibility, data of the interactions were plotted on state space grids in GridWare 1.15a (Lamey et al. 2004), separately for father-child dyads and mother-child dyads. Gridware plots coded emotions (i.e., SACS affect codes) in real-time on state space grids (Fig. 1). A grid represents all possible emotional combinations of a dyad, and each cell on the grid represents a potential emotional state of the dyad. Parents’ coded emotions were plotted on the x-axis and children’s on the y-axis. Any time an emotion changes (of either parent, child, or both), a new point is plotted on the grid and a line is drawn connecting it to the previous point. A trajectory is plotted through the successive points on the grid in the same order as the emotions proceeds in real-time. Hence, a grid represents a sequence of dyadic emotions.

#### Emotional Expressivity

To capture the emotional expressivity, we derived from GridWare the total duration in seconds of dyadic positive affect and dyadic negative affect during father-child and mother-child interactions. Grids were divided into two distinct regions; positive affect included the SACS codes positive affect and validation, and negative affect included the SACS codes distress and anger/disgust. Since two dyad members are rarely expressing emotions simultaneously, which particularly holds for negative emotions (Hollenstein and Lewis 2006), regions were not limited to mutual emotions (Fig. 1). The two measures of expressivity represented the percentage of positive and negative affect as a function of the total duration of the interaction (Van der Giessen et al. 2015). Higher scores indicated that dyads showed more of that particular affect during interactions.

#### Emotional Flexibility

Three indices of dyadic emotional flexibility, which exhibit good reliability and predictive validity (Granic et al. 2003; Hollenstein et al. 2004; Van der Giessen et al. 2015), were derived from GridWare. First, *transitions* (emotional switching) assessed the number of dyadic changes per minute between cells on the state space grid, and it was corrected for differences in total duration of the discussions. Higher values indicated more frequent changes between dyadic emotional states. Second, *dispersion* (emotional repertoire) assesses the spread of dyadic emotional states. Dispersion ranges from 0 to 1, with values close to 1 indicating that behavior was equally distributed across cells and values of 0 indicating that behavior was in only one cell. Higher values indicated a broader dyadic emotional repertoire. Third, *average duration* (emotional rigidity) assessed the mean duration of each dyadic expressed emotion. In contrast to transitions and dispersion, higher values indicated more emotional rigidity of dyads as they tended to remain in dyadic emotions for longer periods of time. Lower values indicated more emotional flexibility of dyads as they tended to remain in dyadic emotions for shorter periods of time.

To create more meaningful measures of emotional flexibility, all measures of emotional flexibility were calculated excluding the mutual neutral cell on the grid (Connell et al. 2011). Mutual neutral affect was most frequently occurring, since this is the default affect code of the SACS coding system. As such, including mutual neutral affect could potentially distort measures of emotional flexibility. Correlations between the three flexibility measures were significant, in the expected direction, and of moderate to high strength.^1^

### Analytic Strategy

Chi-square and *t*-test analyses revealed no differences in gender, age, educational level, and family income between families of whom observational measurements were available (*n*_AD children_ = 113; *n*_non-AD children_ = 35) or not available (*n*_AD children_ = 15; *n*_non-AD children_ = 9). Little's (1988) MCAR Tests were also non-significant and produced normed χ^2^ (χ^2^/*df)* of 1.10 (AD children) and of 0.23 (non-AD children), indicating a good fit between sample scores with and without imputation (Bollen 1989). The flexibility measure average duration and the expressivity measures positive and negative affect of father-child and mother-child dyads showed one extreme univariate outlier. Since extreme outliers could distort multiple imputation and analyses of variance, we recoded these values into the next highest (non-outlier) value (Baraldi and Enders 2010; Tabachnick and Fidell 2007). Missing values were imputed in LISREL 9 using Multiple Imputation techniques (10 imputations) with an EM algorithm, which is recommended as an efficient missing data handling technique (Baraldi and Enders 2010). Imputed data were used in further analyses in SPSS. Analyses were also executed before imputing the data, and without the extreme outliers. Results were similar, indicating that findings are robust. Of note, 6 children in the non-AD group had an AD. When excluding these children from the analyses results were similar. Since our sample was relatively small, we decided to retain these children. Finally, regarding demographic variables between AD and non-AD children were comparable, except that mothers’ and fathers’ educational level was significantly higher for non-AD children than AD children (Table 1).

Group differences in expressivity were examined in SPSS21 using repeated measures ANOVAs, with expressivity type (positive and negative affect) and parents’ gender (mother and father) as within-subjects factors, and group (AD children, and non-AD children) as between-subject factor. Group differences in flexibility were examined using repeated measures ANOVAs, with flexibility type (dispersion, transitions, average duration) and parents’ gender (father and mother) as within-subjects factors, and group(AD children and non-AD children) as between-subject factor. Next, the effects of parental AD on group differences in expressivity and flexibility were analyzed in additional repeated measures ANOVAs, with emotional processes as within-subjects factors, and parental AD (Non-AD child and parent, child AD/non-AD parent, child and parent AD) as between-subjects factor. Of note, in our sample there were no non-AD children who had a father or mother with an AD. Since we were interested in the specific effects of paternal and maternal AD on the dyadic emotional processes during interactions, analyses were performed separately for father-child and mother-child interactions. This way we were able to examine specific effects of paternal AD on father-child expressivity and flexibility, and specific effects of maternal AD on mother-child expressivity and flexibility. Since the three flexibility measures had different scales, analyses were performed with the Z-scores (average duration was reversed). For follow-up comparisons adjusted SIDAK comparisons were used.

## Results

### Effects of Children’s AD

See Table 2 for descriptives of emotional expressivity and emotional flexibility of AD and non-AD parent-child dyads, and Table 3 for the main results of the repeated measures ANOVA’s. There was a main effect of emotional *expressivity* type, indicating that parent-child dyads with and without AD children showed significantly more negative affect than positive affect during interactions. There was a main effect of parents’ gender; mother-child dyads displayed more positive affect as well as more negative affect during interactions than father-child dyads. No differences in emotional expressivity between dyads with and without AD children were found, since no effects of group were found.

**Table 2:** Descriptives of emotional expressivity and emotional flexibility for father-child and mother-child dyads

				AD children (*N* = 128)	
		Non-AD children (*N* = 44)	AD children (*N* = 128)	AD children, non-AD fathers (*N* = 110)	AD children and fathers *(N* = 18)
		*M (SD)*	*M (SD)*	*M (SD)*	*M (SD)*
Father-child dyads					
Negative affect		3.55 (2.69)	5.01 (4.92)	4.79 (4.79)	6.37 (5.62)
Positive affect		2.44 (1.88)	2.69 (2.48)	2.66 (2.34)	2.86 (3.29)
Dispersion		0.65 (.14)	.55 (.22)	0.55 (.23)	0.52 (.18)
Transitions		27.94 (5.80)	19.21 (9.64)	19.41 (9.92)	17.99 (7.84)
Average duration		1.93 (.44)	2.97 (1.49)	2.94 (1.50)	3.15 (1.47)
				AD children (*N* = 128)	
		Non-AD children (*N* = 44)	AD children (*N* = 128)	AD children, non-AD mothers (*N* = 86)	AD children and mothers *(N* = 42)
		*M (SD)*	*M (SD)*	*M (SD)*	*M (SD)*
Mother-child dyads					
Negative affect		5.16 (4.38)	5.72 (5.19)	5.34 (5.31)	6.48 (4.93)
Positive affect		2.64 (2.91)	2.91 (3.08)	2.25 (2.17)	4.24 (4.11)
Dispersion		0.68 (.12)	.62 (.21)	0.62 (.19)	0.63 (.24)
Transitions		26.27 (6.37)	20.70 (9.62)	22.34 (10.36)	17.32 (7.04)
Average duration		2.18 (.66)	3.11 (1.93)	2.74 (1.34)	3.90 (2.61)

Higher values of transitions and dispersion indicates *more* emotional flexibility, while, higher values of average duration indicated *less* emotional flexibility of dyads. For analyses the measure average duration was reversed

*M* Mean, *SD* Standard deviation, *AD* Anxiety Disorder

**Table 3:** Repeated Measures ANOVAs of emotional expressivity and emotional flexibility of parent-child dyads with AD and non-AD children

	*F*	*df* (1), *df* (2)	Partial *η* ^2^
Emotional Expressivity			
Expressivity type	25.56***	1, 170	0.15
Expressivity type * Group	0.85	1, 170	0.01
Gender parent	6.78**	1, 170	0.04
Gender parent * Group	0.72	1, 170	0.01
Expressivity type * Gender parent	3.52	1, 170	0.02
Expressivity type * Gender parent * Group	0.84	1, 170	0.01
Group^1^	2.56	1, 170	0.02
Emotional Flexibility			
Flexibility type	0.92	2169	0.01
Flexibility type * Group	3.89*	2169	0.05
Gender parent	0.45	1, 170	0.00
Gender parent * Group	1.90	1, 170	0.01
Flexibility type * Gender parent	0.06	2, 169	0.00
Flexibility type * Gender parent * Group	0.26	2169	0.00
Group^1^	32.70***	1, 170	0.16

Expressivity types are positive and negative affect. Flexibility types are dispersion, transitions, average duration. Group : 0 = non-AD children, 1 = AD children. Gender parent: 0 = father, 1 = mother. *df* = degrees of freedom, Partial *ƞ* ^2^ = effect size

^1^Result of between-subject effects test

* *p* = 0.05, ** = *p* < 0.01, *** = *p* < 0.001

Regarding emotional *flexibility*, there was a main effect of group; parent-child dyads with AD children showed lower levels of flexibility (dispersion, transitions, and average duration) than parent-child dyads with non-AD children. An interaction effect was found between flexibility type and group. SIDAK comparisons revealed that parent-child dyads with AD children showed a smaller repertoire, *F* (1170) = 5.08, *p* < 0.005, *η* ^2^ = 0.05, switched less between emotions, *F* (1170) = 18.77, *p* < 0.001, *η* ^2^ = 0.17, and displayed emotions longer than parent-child dyads with non-AD children, *F* (1170) = 13.49, *p* < 0.001, *ƞ* ^2^ = 0.12. No differences between father-child and mother-child dyads were found, since no interaction with parents’ gender was found.

### Effects of Parents’ AD

See Table 2 for descriptives of emotional expressivity and emotional flexibility and parental AD, and Table 4 for the main results of the repeated measures ANOVA’s. For emotional *expressivity* of father-child dyads, no significant effect of paternal AD was found. That is, dyads with fathers and children with an AD did not differ in the level of expressivity from father-child dyads with only children (not fathers) with an AD nor from dyads with non-AD children and parents, indicating that paternal AD does not affect emotional expressivity. However, note that only 18 fathers had an AD. There was a significant main effect of maternal AD on the expressivity of mother-child dyads. Follow-up comparisons showed, *F* (2169) = 4.45, *p* = 0.013, *η* ^2^ = 0.05, that when mothers and children had an AD, dyads showed significantly more positive affect as well as more negative affect during interactions than when dyads with AD child and non-AD mothers (*p* = 0.014), and compared to non-AD dyads (*p* = 0.006). There was no difference between non-AD mother-child dyads and mother-child dyads with AD children and non-AD mothers (*p* = 0.996). These results indicate that maternal AD increases emotional expressivity of parent-child dyads.

**Table 4:** Repeated Measures ANOVAs of the effects of parental AD on emotional expressivity and emotional flexibility

	*F*	*df* (1), *df* (2)	Partial *ƞ* ^2^
Father-Child Dyads Emotional Expressivity			
Expressivity type	19.18***	1, 169	0.10
Expressivity type * Parental AD	1.44	2, 169	0.02
Parental AD ^1^	3.05	2, 169	0.04
Father-Child Dyads Emotional Flexibility			
Flexibility type	0.12	2, 168	0.00
Flexibility type * Parental AD	1.98	4, 169	0.00
Parental AD^1^	15.34***	2, 169	0.15
Mother-Child Dyads Emotional Expressivity			
Expressivity type	31.37***	1, 169	0.16
Expressivity type * Parental AD	0.34	2, 169	0.01
Parental AD^1^	4.46*	2, 169	0.05
Mother-Child Dyads Emotional Flexibility			
Flexibility type	0.28	2, 168	0.00
Flexibility type * Parental AD	3.42**	4, 169	0.04
Parental AD^1^	17.87***	2, 169	0.11

Expressivity types are positive and negative affect. Flexibility types are dispersion, transitions, average duration. Parental AD: 0 = non-AD parent and child, 1 = child anxiety disorder, non-AD parent, 2 = child and parental anxiety disorder. *df* = degrees of freedom, Partial *ƞ* ^2^ = effect size

^1^Result of between-subject effects test

* = *p* < 0.05, ** = *p* < 0.01, *** = *p* < 0.001

For emotional *flexibility* of father-child dyads, a significant main effect of paternal AD was found. However, follow-up comparisons showed, *F* (2169) = 15.34, *p* < 0.001, *η* ^2^ = 0.15, that dyads with AD fathers and AD children did not differ in the amount of emotional flexibility during interactions from dyads with only children (not fathers) with an AD (*p =* 0.772). It was only found that father-child dyads with AD children and non-AD fathers (*p* < 0.001) , and dyads with AD fathers and children (*p* = 0.001) showed less emotional flexibility compared to non-AD father-child dyads. These results indicate that paternal AD does not further reduce emotional flexibility of parent-child dyads.

There was a significant main effect of maternal AD on the flexibility of mother child dyads. Also, there was a significant interaction effect between maternal AD and flexibility type. For transitions follow-up comparisons showed, *F* (2169) = 11.42, *p* < 0.001, *η* ^2^ = 0.12, that AD mother-child dyads showed less transitions compared to dyads with AD children and non-AD mothers (*p* = 0.008), and compared to non-AD mother-child dyads (*p* < 0.001). Dyads with AD children and non-AD mothers, also showed less transitions than non-AD mother-child dyads (*p* = 0.004). For average duration follow-up comparisons showed, *F* (2169) = 12.55, *p* < 0.001, *η* ^2^ = 0.13, that AD mother-child dyads showed a higher average duration of emotions compared to dyads with AD children and non-AD mothers (*p* < 0.001) and compared to non-AD mother-child dyads (*p* < 0.001). Dyads with AD children and non-AD mothers, also showed higher average duration than non-AD mother-child dyads (*p* = 0.004). For dispersion we did not find a difference between the groups, *F* (2169) = 1,76, *p* = 0.175, *η* ^2^ = 0.02. These results indicate that when mothers and children have an AD (AD dyads) this significantly reduces the number of emotional changes, and increases the emotional duration of mother-child dyads compared to when only children, not mothers, have an AD and compared to non-AD mother-child dyads. However, maternal AD did not affect the emotional repertoire of mother-child dyads.

### Effects of Gender and Age

Given potential age and gender mean differences we explored whether our findings varied by age and gender. To be able to include age as a between-subjects factor in our exploratory analyses, we divided our sample into two age groups based on their respective developmental period, namely children (8 to 12 years) and adolescents (13 to 18 years). Preliminary *t*-tests revealed no differences between children and adolescents in levels of dyadic emotional expressivity and flexibility. There was one exception; mother-child dyads with children showed more negative affect than mother-child dyads with adolescents, *F* (1, 170) = 5.73, *p* = 0.018, *η* = 0.03. Regarding children’s gender, preliminary *t*-tests revealed no consistent differences between boys and girls in levels of emotional flexibility and expressivity of mother-child and father-child dyads. It was only found that mother-daughter dyads showed more negative affect, *F* (1, 170) = 13.92, *p* < 0.001, *η* = 0.08, and less transitions , *F* (1, 170) = 11.07, *p* < 0.001, *ƞ* = 0.06. than mother-son dyads. Therefore, since no consistent age and gender mean differences were apparent and also because of limited power we did not control for children’s age and gender in our main analyses. Nevertheless, as an extra check we explored post hoc whether our results varied by children’s age or gender. This was not the case, indicating that neither parents’ and children’s gender nor children’s age affected differences in emotional expressivity and flexibility between parent-child dyads with and without AD children. However, since our sample size was not large enough for adequately testing gender and age differences, caution is warranted when interpreting these exploratory results. An overview of the results of these exploratory analyses are available upon request from the first author.

## Discussion

This observational study examined differences in emotional expressivity and emotional flexibility of parent-child dyads with AD children and non-AD children, the effects of parental AD on expressivity and flexibility of dyads, and differences between father-child and mother-child dyads. Results showed (1) no differences in emotional expressivity between parent-child dyads with and without AD children, (2) less emotional flexibility in parent-child dyads (i.e., both father-child and mother-child dyads) with AD children than in parent-child dyads with non-AD children, and (3) more emotional expressivity and less emotional flexibility in mother-child dyads with AD mothers and AD children than in mother-child dyads with only AD children (not mothers) or in dyads with non-AD children and mothers.

In contrast with our expectations, parent-child dyads with AD children did not express less positive affect or more negative affect during conflict interactions than dyads with healthy children. This contradicts earlier studies (Hudson et al. 2008; Suveg et al. 2005, 2008) showing deficits in parents’ and children’s emotional expressivity during parent-child interactions with AD children. Since none of these studies investigated emotional expressivity as a real-time sequence of dyadic emotions, one explanation for our results might be that the role of parents’ and children’s individual emotions in child anxiety has been overestimated. During social interactions emotional expressions reflect what goes on between individuals; parent and child dynamically and reciprocally alter their emotions with respect to the ongoing and anticipated emotions of each other (Butler 2011; Fogel 1993; Hinde 1997; Morelen and Suveg 2012). Emotional expressiveness during interactions, therefore, might not be fully understood by considering emotions of parents and children in isolation. Another explanation might be that individual expressivity and dyadic expressivity might provide unique and different insights into emotional processes during parent-child interactions, which might also be differently related to child anxiety. An exploratory factor analysis showed that individual and dyadic measures of parent-infant interactions loaded onto separate factors (Moore et al. 2013), indicating that measures of individual and dyadic expressivity seem to be conceptually and quantitatively different. Future research clarifying micro-level emotional expressions of parents and children is essential to comprehend and target recurring individual and dyadic emotional patterns that are associated with children’s AD.

Findings also revealed that father-child dyads, with and without AD children, displayed less positive affect and less negative affect during conflict interactions than mother-child dyads. Previous research has shown that during parent-child conversations about past emotional experiences, fathers talked less about emotional aspects of the experiences and used less emotion words than mothers (Fivush et al. 2000). Additionally, children tend to have more interpersonal conflicts with mothers than with fathers (Branje et al. 2012), which might make the conflict interactions somewhat more confrontational and relevant for mother-child than father-child dyads. As such, results extend earlier studies by showing that also on a dyadic level father-child dyads in general are less emotionally expressive in a conflict situation than mother-child dyads. Furthermore, both father-child and mother-child dyads, with and without AD children, displayed more negative than positive affect during the interactions. Thereby it seems that our conflict interactions were indeed confrontational for parent and child, and induced more dyadic negativity than dyadic positivity.

In line with dynamic systems theory (Butler 2011; Fogel 1993; Granic 2005), and earlier studies (e.g., Van der Giessen et al. 2015), this study emphasizes that the ability of parent-child dyads to flexibly move in and out of emotions (e.g., emotional flexibility or affective variability) provides relevant information about the nature of dynamic parent-child conflict interactions as it sets apart AD from non-AD parent-child dyads. Parent-child dyads with AD children showed less emotional flexibility by displaying a smaller repertoire of emotions, switching less between emotions, and remaining in emotions for longer periods of time compared to dyads with non-AD children. Parent-child dyads with AD children were less able to adequately manage positive and negative emotions during interactions than healthy dyads. Results seem to suggest that the inhibited and stereotyped emotional responses, high levels of negative experiences, and hyperarousal associated with anxiety disorders of children affected the dyadic emotional dynamics as parents and children interact. Our work may help clinicians as well as families with children with an AD understand what (in)adequate dyadic emotion regulation is, and how to adapt dyadic emotional patterns accordingly. It could be argued that parent-child dyads with AD children should be guided during interactions in shifting between a wide variety of positive and negative emotions with relative ease. Nevertheless, intervention studies are needed to investigate whether improvements in child anxiety may also benefit dyadic emotional flexibility.

Current results may imply that dyadic emotional flexibility might be a better indicator of problematic parent-child emotional processes when comparing AD and non-AD children than dyadic emotional expressivity. Although it is often thought that negative emotions should be reduced, or even suppressed, and positive emotions should be encouraged during parent-child interactions with AD children (Waite et al. 2014), this study showed that, at least at a dyadic level, it might be desirable for parent-child dyads to flexibly express a broad range of both positive and negative emotions. This is in line with propositions of emotion theorists (Gross 2007; Izard 2009), suggesting that all emotions are important to express for healthy functioning. For example, some degree of negativity of parent-child dyads during conflict interactions is appropriate, as long as it is managed effectively. Dyads getting stuck in emotions seems to be more problematic (Granic et al. 2007; Houben et al. 2015). Since, differences between father-child and mother-child dyads were not found, more emotional flexibility of father-child as well as mother-child dyads seems to be the hallmark of healthy emotional functioning. Altogether, current work expands the emerging evidence that dyadic emotional inflexibility is associated with psychopathology, including anxiety disorders. Further research should compare emotional flexibility of parent-child dyads in different clinical groups (e.g., depression, anxiety, conduct disorder), as this would provide an even richer understanding of whether different type of disorders are characterized by similar (or distinctive) dyadic emotion dynamics during interactions, and such knowledge might inform and facilitate prevention and intervention.

Maternal AD affected levels of emotional expressivity and emotional flexibility of mother-child dyads with AD children. This means that when both mothers and children had an AD, dyads expressed more positive and negative affect, displayed emotions for longer periods of time, and switched less between emotions compared to dyads where only children, not mothers, had an AD and compared to dyads with non-AD children and mothers. Although AD mother-child dyads were quite expressive while discussing a difficult conversation topic, they tended to get stuck in these emotions. Thus, it seems to affect emotional flexibility of dyads when both mother and child have an AD. Reciprocal exchanges between AD mothers and AD children might escalate the experience of negative and positive emotions, making it difficult for dyads to return to the optimal bounds of emotional functioning, thereby getting stuck in dyadic emotions (Butler and Randall 2013). Nevertheless, current finding that dyads with AD mothers and children were more emotionally expressive is in contrast with earlier studies showing that individual emotional expression is reduced or suppressed during interactions in families with AD (e.g., Suveg et al. 2005). There are several potential explanations. First, as mentioned before, differences may be due to measuring expressivity on an individual or a dyadic level (Moore et al. 2013). Second, it might also be the result of the different conversations topics used between different studies; our conflict interaction might not affect expressivity in the same way as a discussion about a recently experienced emotion by the child does (e.g., Suveg et al. 2008). Third, and related, the isolated focus of earlier studies on the amount of positive and negative emotions might overlook that (in)adaptive levels of expressiveness depend on the emotional demands of the context. For example, a certain amount of suppression of emotions might be valuable when solving a difficult cognitive or social task, while this might be more problematic when trying to work through an interpersonal conflict. Together, we think that our results again seem to advocate that problematic emotional interaction patterns of mothers and children are best captured by examining dyadic emotional flexibility in real-time (Butler 2011; Moore et al. 2013; Van der Giessen et al. 2015). Of note, we were unable to examine the effect of maternal AD (and paternal AD) on interactions of parent-child dyads with non-AD children. Research on depression has found that maternal depression was associated with more negative and rigid dyadic interactions of mothers and their non-depressed adolescents (Connell et al. 2011). Future research should therefore examine with larger samples if maternal AD (and paternal AD) is also associated with dyadic inflexibility in interactions with typically developing children.

Although a similar pattern of more expressivity and flexibility was evident for dyads with AD fathers, no significant effects of paternal AD on dyadic expressivity and flexibility were found. These results might indicate that dyads with both fathers and children with an AD did not differ with regard to levels of expressivity and flexibility from dyads in which only children had an AD. However, there are several potential explanations why no effects were found. First, the small number of fathers with an AD (*n* = 18) in our sample, may have prevented us from detecting group differences. Second, father-child dyads already displayed less emotional expressivity and less emotional flexibility than mother-child dyads. Non-significant effects might be due to a floor effect; father-child dyads with AD children may already score at the lower end of the scale. Third, fathers’ AD might have a negligible effect on dyadic expressivity or flexibility, but affect fathers’ individual parenting behaviors, such as their challenging parenting behavior (Bögels and Phares 2008). Replication or our findings with larger samples is necessary to draw more firm conclusions about the effects of paternal as well as maternal AD.

Our exploratory analyses showed that results did not vary by gender and age of children. Despite the fact that gender and age impact children’s own emotion regulation skills, with girls being more expressive and regulated than boys, and with older children showing more sophisticated emotion expression and better emotion management (Morris et al. 2007), our exploratory results seem to suggest that dyadic emotional processes of AD and non-AD parent-child dyads do not differ by gender and age. Anxiety disorders of children and parents might be related to inflexible emotion regulation in similar ways for parent-daughter dyads and parent-son dyads from middle childhood to adolescence. However, since the current study had limited power to detect age and gender differences, future studies should examine whether our exploratory findings hold up with larger samples.

The present study extends previous research on dyadic emotional processes during parent-child interactions related to children’s AD. Although the current study has a number of important strengths, such as the observational design, the comparison of AD and non-AD children, the examination of real-time dyadic emotions using innovative state space grid analyses, and the inclusion of father-child and mother-child dyads, several limitations should also be noted and addressed in future research. First, this study focused on emotional processes that occur within conflict interactions. Yet, emotional demands are different across contexts, and the merit of emotional expressivity and flexibility might depend on the specific social context. Future research should address the role of emotional expressivity and flexibility of dyads across different contexts. Second, since this was a cross-sectional study, we were unable to infer causal relationships. A prospective longitudinal design could elucidate whether emotional rigidity of dyads precedes children’s AD or vice versa. Fourth, the size group with non-AD children was relatively small, and we also had limited power to adequately detect effects of parental AD, gender, and age. Examining these group differences with larger samples is necessary to increase our understanding of the role of dyadic emotional processes for child anxiety.

The current observational study represents a novel and important contribution to the current literature by showing that dyadic emotional flexibility, but not dyadic emotional expressivity, was lower in parent-child dyads with children with an AD than dyads with typically developing children. Hence, findings add to the growing acknowledgement (Butler 2011; Granic 2005; Houben et al. 2015) that a focus on the real-time dynamic nature of emotions, particularly dyadic emotional flexibility, during parent-child interactions is important for understanding anxiety disorders. In addition to child AD, particularly maternal AD should also be accounted for when examining dyadic emotional processes. Thus far, including a parent or family component in cognitive behavioral therapy for child AD had no added value to treatment outcome (e.g., Reynolds et al. 2012). These family-based therapies for child anxiety primarily aimed to improve communication patterns by reducing negative affect (hostility and rejection) and increasing positive affect (warmth and autonomy), particularly of parents. However, our work seems to indicate that intervening in emotion dynamics at a dyadic level by teaching and encouraging parent-child dyads to engage together in flexible emotion behaviors might be more effective. Helping dyads to avoid emotional rigidity may be a valuable goal for intervention.

## Footnotes

1 Correlations can be obtained from the first author.
